# Restructuring the Heart From Failure to Success: Role of Structural Interventions in the Realm of Heart Failure

**DOI:** 10.3389/fcvm.2022.839483

**Published:** 2022-04-20

**Authors:** Devika Kir, Mrudula Munagala

**Affiliations:** Department of Cardiology, University of Miami Miller School of Medicine, Jackson Memorial Hospital, Miami, FL, United States

**Keywords:** transcatheter therapies, structural interventions, heart failure, functional valvular regurgitation, robotic sleeves, inter-atrial shunt, ventricular restorative devices

## Abstract

Heart failure through the spectrum of reduced (HFrEF), mid-range (or mildly reduced or HFmEF), and preserved ejection fraction (HFpEF), continues to plague patients' quality of life through recurrent admissions and high mortality rates. Despite tremendous innovation in medical therapy, patients continue to experience refractory congestive symptoms due to adverse left ventricular remodeling, significant functional mitral regurgitation (FMR), and right-sided failure symptoms due to significant functional tricuspid regurgitation (FTR). As most of these patients are surgically challenging for open cardiac surgery, the past decade has seen the development and evolution of different percutaneous structural interventions targeted at improving FMR and FTR. There is renewed interest in the sphere of left ventricular restorative devices to effect reverse remodeling and thereby improve effective stroke volume and patient outcomes. For patients suffering from HFpEF, there is still a paucity of disease-modifying effective medical therapies, and these patients continue to have recurrent heart failure exacerbations due to impaired left ventricular relaxation and high filling pressures. Structural therapies involving the implantation of inter-atrial shunt devices to decrease left atrial pressure and the development of implantable devices in the pulmonary artery for real-time hemodynamic monitoring would help redefine treatment and outcomes for patients with HFpEF. Lastly, there is pre-clinical data supportive of soft robotic cardiac sleeves that serve to improve cardiac function, can assist contraction as well as relaxation of the heart, and have the potential to be customized for each patient. In this review, we focus on the role of structural interventions in heart failure as it stands in current clinical practice, evaluate the evidence amassed so far, and review promising structural therapies that may transform the future of heart failure management.

## Introduction

**Figure 1:**
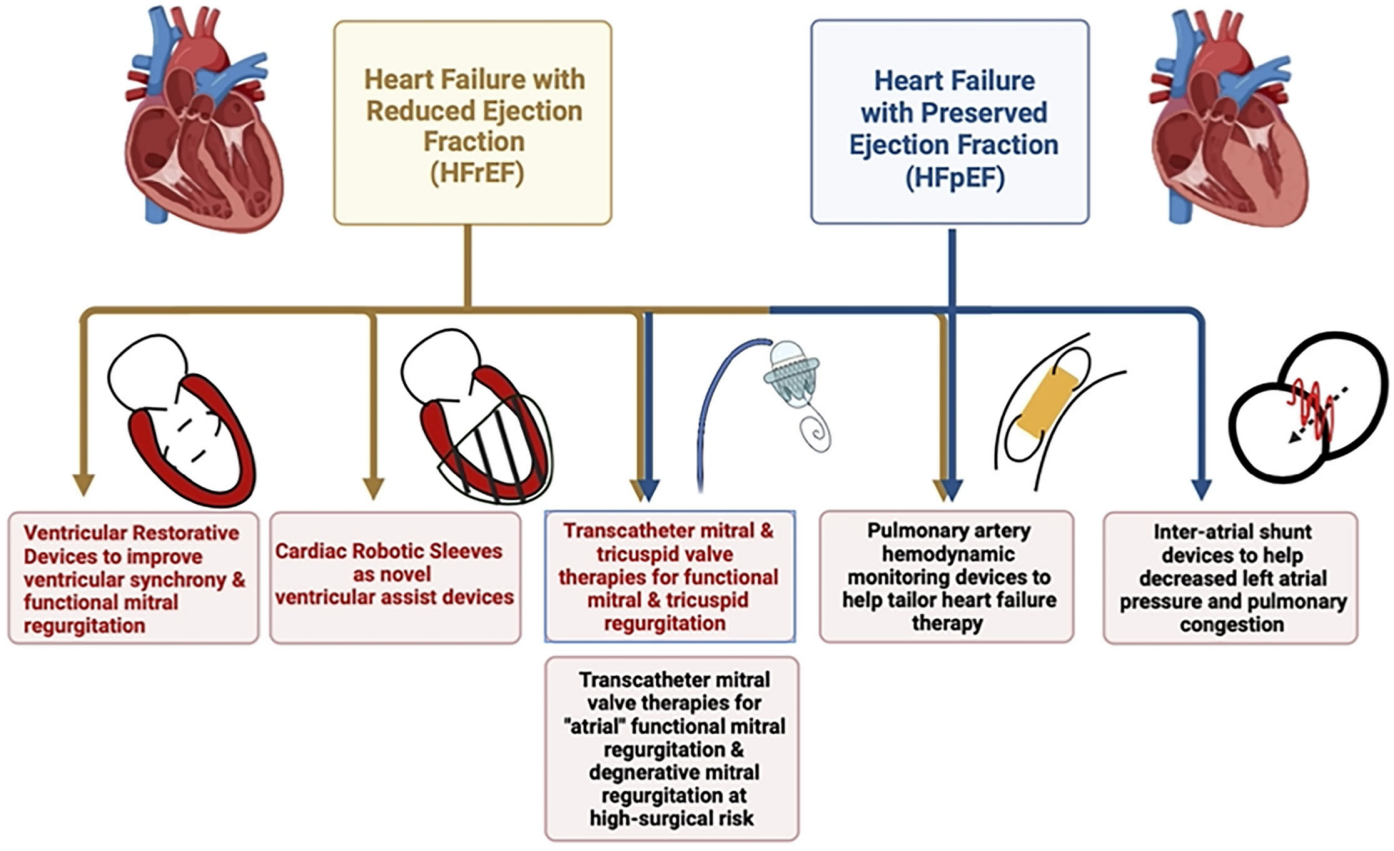
Schematic diagram highlighting the role of different structural interventions in heart failure with reduced and preserved ejection fraction.

Despite tremendous advances in medical therapy and revascularization techniques, heart failure continues to be a growing global epidemic– the prevalence of global heart failure doubled from 33.5 million in 1990 to 64.3 million in 2017 (1). According to American Heart Association 2021 statistics, with our aging population, the prevalence of heart failure is projected to increase by 46% from 2012 to 2030 and would affect >8 million Americans or nearly 3% of the population 18 years or older (2). Heart failure continues to be the foremost cause of hospitalization in the elderly that leads to high mortality, morbidity, and economic burden across the spectrum of heart failure with reduced (HFrEF), mid-range (or mildly reduced or HFmEF), and preserved ejection fraction (HFpEF) (3). Regardless of the ejection fraction, hospitalization for heart failure exacerbation has been a reliable predictor of recurrent admissions and cardiovascular death (2). Although sweeping progress has been made in the realm of HFrEF management, the HFpEF domain is yet to meet with similar fortune. In addition, despite optimal medical management of primary cardiac pathology such as ischemic heart disease and/or cardiomyopathy, patients can still experience refractory congestive symptoms due to the progression of secondary valvular disease (tricuspid and/or mitral regurgitation). Tricuspid regurgitation (TR), especially secondary regurgitation, has been a trifled valvular pathology that has gained some attention in recent times as the milieu of chronic venous congestion and its detrimental effect on end-organ function is better understood (4). These patients are not usually favored for surgical valvotomy or valvular replacement due to perceived high surgical risk that is attributed to poor functional status, underlying disease process, recurrent exacerbations, and significant comorbidities. Hence, in the last decade, structural interventions aimed at improving functional valvular regurgitation unlatched new frontiers for those high-risk patients in whom surgery is not feasible. In addition to helping tackle valvular disease percutaneously, novel structural devices are being developed to help monitor pulmonary pressures in a real-time fashion, to effect left ventricular reverse remodeling and improve effective stroke volume, and lastly, there is pre-clinical data supportive of soft robotic cardiac sleeves that serve to improve cardiac function and can have the potential to be customized for an individual patient (Figure 1). In this review, we emphasize the role of structural interventions in heart failure as it stands in current clinical practice, evaluate the evidence amassed so far, and review promising structural therapies that may transform the future of heart failure management.

## Structural Interventions Targeting Valvular Heart Disease and Heart Failure

### Mitral Regurgitation

Moderate or severe functional or secondary MR accompanies heart failure in about one-third of the patients and is mediated by mal-coaptation of the valve leaflets due to left ventricular remodeling and dysfunction secondary to ischemic and/or non-ischemic etiologies or due to annular dilatation in patients with long-standing atrial fibrillation or “atrial FMR” (5, 6). Mechanisms causing FMR include an increase in tethering forces causing tenting of the valve due to left ventricular dilation and increased sphericity, displacement of the papillary muscles, and annular dilatation. Further, there is a reduction in mitral valve closure during systole due to impaired contractility and a reduction in mitral annular movement (6). MR further strains the dysfunctional ventricle by causing volume overload and is independently predictive of increased mortality and other adverse heart failure outcomes (7–9). As most of these patients with advanced heart failure are unfavorable or unpropitious surgical candidates, there has been tremendous enthusiasm and research in the management of FMR through minimally invasive or percutaneous strategies. One such method relies on replication of the “Alfieri stitch” that approximates the anterior and posterior leaflets together to reduce mitral regurgitation percutaneously using trans-septal access– Transcatheter Edge-to-Edge Repair (TEER) using the Mitraclip™ (Abbott) or PASCAL™ (Edwards Lifesciences) devices (available only in Europe). Based on the reduction in heart failure hospitalizations and mortality rates with TEER that were noted in the COAPT trial, TEER has been given a class IIA recommendation for patients with HFrEF and chronic, severe FMR with persistent heart failure symptoms [New York Heart Association (NYHA) class II-IV] despite optimization of medical therapy as long as the mitral valve anatomy is conducive for the procedure, LVEF is between 20 and 50%, with left ventricular end-systolic dimension (LVESD) ≤ 70 mm and pulmonary artery systolic pressure is ≤ 70 mm Hg (10, 11). Another contemporary trial that evaluated the role of TEER for secondary mitral regurgitation–MITRA-FR, however, failed to show any difference in clinical outcomes with percutaneous mitral valve repair in a similar population (12). Given subtle differences in the inclusion criteria, patients enrolled in the COAPT trial had more severe valvular heart failure with a lesser degree of dysfunctional/dilated myocardium, better medical and device therapy and better optimization of MR with a larger number of Mitra-clips deployed per patient when compared to patients enrolled in the MITRA-FR trial (13). This discrepancy between the results noted in COAPT and MITRA-FR highlighted the importance of assessment of the severity of mitral regurgitation in proportion to the severity of left ventricular dysfunction and introduces the concept of “proportionate MR as noted in MITRA-FR” and “disproportionate MR as noted in COAPT.”

Not all mitral valve anatomies are ideal for performing a TEER and hence alternative mitral valve repair strategies are needed. One such device is a self-expandable nitinol ring that can be implanted in the coronary sinus through the right jugular vein– the Carillon™ Mitral Contour System– this device relies on the close relationship between the coronary sinus and the mitral valve annulus to effect an indirect annuloplasty (14). Advantages of this device include ease of venous access without the requirement of trans-septal puncture and it gives the operators a different mechanism to address FMR by targeting annular dilatation and does not preclude other valvular therapies. Circumflex artery has been shown to cross between the coronary sinus and the mitral valve annulus in about 80% of the patients and depending on the patient's anatomy, it may preclude this procedure in some patients given the inherent risk of impingement or compression of the artery when the annuloplasty band is deployed (15). Multiple trials (AMADEUS, TITAN, and TITAN II) have established the safety and feasibility of successive iterations of the Carillon™ device for FMR (16–18). REDUCE-FMR randomized patients with severe FMR to device therapy compared to a sham control and showed low device failure rates (14%) and complications, with a reduction in regurgitant volume and left ventricular remodeling and improvement in functional outcomes at 1 year (19). The largest sham-controlled trial to evaluate the safety and efficacy of Carillon™ trial in patients with symptomatic FMR (at least mild severity)—the EMPOWER trial– is currently recruiting patients (20). This trial is unique in including patients with mild FMR to assess any difference in clinical and functional outcomes with device therapy.

Another device that targets annular dilatation is a minimally invasive, catheter-delivered direct annuloplasty ring– the Cardioband™ valve reconstruction system (Edwards Lifesciences) (21). This implant is anchored along the posterior mitral valve annulus using steel anchors through a trans-septal approach under fluoroscopic and echocardiographic guidance and involves adjustment of the annular size through a specific size adjustment tool (22). Similar to the TEER devices, an adequate septal length is needed for trans-septal access, and similar to the Carillon devices, specific anatomical issues with the crossing of the circumflex artery can preclude this therapy. A European multi-center study established feasibility with a favorable safety profile for the Cardioband™ device in 31 patients with symptomatic moderate to severe FMR with sustained improvement in annular size, mitral regurgitation, and functional outcomes at 7 months follow-up (23). ACTIVE is an ongoing randomized controlled trial to evaluate the safety and efficacy of the Cardioband™ device compared to medical therapy alone for patients with clinically significant and symptomatic FMR with a long follow-up period of 5 years (24). Mitralign™ (Massachusetts) is another direct annuloplasty device that uses radiofrequency energy to penetrate two pairs of pledget sutures through the mitral annulus tissue from the left ventricular aspect into the left atrium– the annulus and mitral regurgitation are reduced by cinching the sutures. Feasibility study for Mitralign™ in 71 patients with moderate-severe FMR showed technical success in 70% of the patients enrolled with a successful reduction in MR at 6 months in 50% of the patients (25). Mistral™ (Mitralix Ltd.) is a spiral-shaped investigational device that reduces FMR by grasping the chordae tendineae and improving leaflet coaptation– a feasibility study (MERIT) is currently enrolling patients with severe FMR (26).

While these Transcatheter Mitral Valve Repair (TMVr) techniques offer promising results for patients with FMR, some patients have unfavorable mitral valve anatomy (such as calcified mitral annulus) for percutaneous repair and may experience complications or failure with significant residual or recurrent MR despite TMVr (27). Transcatheter Mitral Valve Replacement (TMVR) options are hence being explored aggressively to develop viable alternatives in such patient populations. Owing to the saddle-shaped, dynamic mitral valve annulus and proximity to the left ventricular outflow tract with potential for obstruction, the development of a prosthesis in this location has its unique challenges. A number of prostheses with different designs have been successfully implanted in humans– the CardiAQ™, the Intrepid™, the HighLife™, the Tiara™, the Tendyne™, the AltaValve™, the EVOQUE™, the SAPIEN M3™, and the Cardiovalve™ (Table 1) (44). Because of technical challenges with the positioning of the device and co-axial prosthesis alignment that involves a 90° turn after crossing the interatrial septum, most of the devices were developed to be deployed through trans-apical access except for the EVOQUE™, the SAPIEN M3™, the Intrepid™, and the Cardiovalve™, which have been successfully deployed in patients through a trans-septal approach utilizing transfemoral vascular access. Among these TMVR devices, the longest follow-up data is available for the Tendyne™ valve– in 100 patients with severe, symptomatic FMR at high-surgical risk without significant valvular or annular calcification, deployment of the Tendyne™ valve was associated with procedural success, reduction in heart failure hospitalizations and persistent reduction in MR without structural degeneration at 2-year follow-up (36). While TMVR offers a more durable reduction in FMR, it does involve a more invasive approach which can lead to increased bleeding complications and a longer hospital course in the frail, elderly population. With the trans-septal approach, the resulting large iatrogenic ASD with TMVR can precipitate volume overload and heart failure decompensations and can cause a right-left shunt with hypoxemia in this high-risk patient population with pulmonary hypertension– the role of closure of this ASD and the timing of the closure is not clear and closure devices can impede access for any future procedures needing trans-septal access. SUMMIT is an ongoing randomized controlled trial comparing TEER with the Tendyne™ valve for patients with symptomatic severe FMR and is also going to evaluate the Tendyne™ valve for patients with significant annular calcification– this trial would help guide patient selection for TMVr vs. TMVR in the future (37).

**Table 1:** Descriptive analysis of the different Transcatheter Mitral Valve Replacement devices with human experience.

**Valve name**	**Valve structure**	**Access for deployment (sheath size)**	**Level of evidence**
CardiAQ™ (Edwards Lifesciences)	Nitinol, self-expanding trileaflet bovine pericardial valve (30-mm) with circumferential anchors on the atrial and ventricular side	Trans-apical, trans-septal (31 Fr)	Early Feasibility Study (RELIEF) has been withdrawn due to high 30-day mortality rates (28). This device has been redesigned as the EVOQUE™.
Intrepid™ (Medtronic)	A self-expanding, nitinol frame with a dual ring design creates a “champagne-cork-like effect” for anchoring. The inner stent frame includes a 27-mm trileaflet bovine pericardium valve.	Trans-apical and Trans-septal (35 Fr)	Early experience of 50 patients- technical success (98%) (29). Ongoing single-arm APOLLO trial for patients with severe, symptomatic MR, including patients with MAC utilizing trans-apical access (30). A feasibility study involving trans-femoral access in 15 patients with severe symptomatic mitral regurgitation (mostly primary MR) at high-surgical risk showed technical success in 93% of the patients with trace/no MR or paravalvular leak, no deaths or strokes at 30 days follow-up (31).
HighLife™ 2-component system (HighLife Medical)	“Valve-in-ring”- Nitinol, self-expanding 31- or 28-mm trileaflet bovine bio-prosthesis is used with a sub-annular implant that is deployed through trans-septal access.	Trans-apical (39 Fr), Trans-septal access for the ring (18 Fr)	Feasibility study for severe, symptomatic MR is ongoing for the 31 mm trans-apical implant−5 patients have been recruited so far (32) and the 28-mm trans-septal TMVR (33).
Tiara™ (Neovasc Inc.)	Self-expanding, nitinol, D-shaped frame, trileaflet, bovine pericardial valve (35- or 40-mm), the frame has three ventricular anchors.	Trans-apical (32/36 Fr)	This device is currently being evaluated in feasibility (TIARA-I) (34) and a safety and performance clinical study (TIARA-II) (35).
Tendyne™ (Abbott Laboratories)	Valve is fully repositionable and retrievable. Trileaflet, a porcine pericardial valve on a self-expanding, nitinol double-frame with an epicardially fixed apical pad. An atrial cuff further helps to anchor the valve.	Trans-apical (34 Fr)	A feasibility study of 100 patients with a 2-year follow-up shows technical success in 97% of the patients. Thirty-nine percent all-cause mortality at 2 years (36). SUMMIT trial to evaluate TEER vs. Tendyne™ and also evaluate Tendyne™ in patients with MAC (37).
AltaValve™ (4C Medical Technologies Inc.)	First TMVR implanted in a supra-annular position to help minimize LVOTO. Trileaflet bovine valve (27 mm) in a self-expanding spherical nitinol stent.	Trans-apical (32 Fr)	Ongoing early feasibility study (38). The trans-septal access system is under development.
EVOQUE™ (Edwards Lifesciences)	Redesigned CardiAQ™ valve (available in 44- and 48-mm sizes) with a lower profile for trans-septal delivery, lower ventricular projection to minimize LVOTO.	Trans-septal (28 Fr)	First-in-human experience (14 patients) with good technical success (93%) (39). Ongoing early feasibility study (40).
SAPEIN M3™ (Edwards Lifesciences)	Balloon-expandable, trileaflet bovine pericardial valve (29-mm) on a nitinol stent, based on the SAPIEN 3 TAVR system. Nitinol dock encircles the chordae tendineae securing the valve in place.	Trans-septal (20 Fr)	First-in-human experience (10 patients) with good technical success (90%) (41). Ongoing early feasibility study (ENCIRCLE) (42).
Cardiovalve™ (Cardiovalve Ltd.)	Trileaflet, 3-scallop shaped bovine pericardial valve (40–50 mm) in a self-expanding nitinol stent with 24 ventricular anchors.	Trans-septal (28 Fr)	Ongoing early feasibility study (AHEAD) (43).

*Fr, French; MR, Mitral Regurgitation; MAC, Mitral Annular Calcification; LVOTO, Left Ventricular Outflow Tract Obstruction; TAVR, Transcatheter Aortic Valve Replacement*.

### Tricuspid Regurgitation

In 2005, ~1,600,000 patients were identified to have moderate-severe TR, however, only <8,000 of these patients underwent tricuspid repair or replacement (45). Primary TR is relatively uncommon (<10%) and is mostly mediated by left-sided valvular disease, pulmonary hypertension, left- and right-sided cardiomyopathies (46). TR has been a neglected pathology; however, it is clear that patients with moderate or higher severity of TR have worse outcomes with higher mortality rates, even after adjusting for pulmonary pressures, right ventricular function, and left ventricular ejection fraction (47, 48). FMR and FTR commonly coexist. In patients undergoing surgery for left-sided valvular disease, it is a class I recommendation to intervene on severe concomitant TR or moderate TR with a dilated annulus, however, no such guidelines exist for transcatheter therapies. A contemporary comparison of two cohorts of patients with concomitant severe functional MR and TR, patients who underwent transcatheter repair of the mitral and tricuspid pathology in the international TriValve registry had improved 1-year survival rates compared to TMVr alone in the German TRAMI registry (49). TMVr has been shown to improve TR in a third of these patients with secondary TR, however, persistent moderate-severe TR is common and is predictive of adverse patient outcomes (50). Persistent significant TR creates volume overload and strains the right ventricle furthering right ventricular dysfunction, tricuspid annular dilatation, which then leads to worsening of TR, thereby creating a vicious downward spiral. If uncorrected, persistent TR can lead to diuretic resistance, and multi-organ failure with renal injury and cirrhosis. Given the high surgical mortality in patients with secondary TR, the development of transcatheter therapies for tricuspid valve repair and replacement has been an area of active research. There is evidence for worse clinical outcomes for tricuspid valve surgery or transcatheter tricuspid valve intervention in patients with evidence of cardio-hepatic syndrome (51, 52). This calls for timely intervention for FTR before organ failure ensues. Patients with FTR are commonly anticoagulated for concomitant atrial fibrillation which makes scoring systems that rely on INR levels like the MELD (Model for End-Stage Liver Disease) score unreliable for assessment of liver dysfunction. Novel scores for assessing liver dysfunction are being actively researched– one such score that factors in patient's age, evidence of renal dysfunction, diuretic resistance and hepatic dysfunction– the TRISCORE– was recently validated as a mortality predictive tool for patients undergoing isolated tricuspid valve surgery (53). The complex anatomy of the tricuspid valve with an asymmetric, large annulus, proximity to important structures like the right coronary artery and AV node and, difficulty in imaging with subjective criteria for grading of TR severity has made the development of these therapies further challenging.

Treatment strategies for Transcatheter Tricuspid Repair (TTVr) include TEER using the Triclip™ (Abbott) and PASCAL™ (Edwards Lifesciences) systems, direct annuloplasty with Cardioband™ (Edwards Lifesciences), Tricinch™ (4Tech Cardio Ltd.), Trialign™ (Mitralign Inc.) and the Mistral™ (Mitralix Ltd.) device which effects a reduction in TR through grasping and inward pulling of the chordae tendineae (Table 2). Among these devices, TEER and annuloplasty with Cardioband™ and the Trialign™ devices are based on the known mitral valve repair techniques–owing to ease of use and familiarity, TEER has been the most commonly employed therapy for the tricuspid valve as well (60). TTVr devices were being used initially on a compassionate basis– Triclip™, PASCAL™, and the Cardioband™ devices were recently granted Conformité Européenne (CE) approval. These repair devices can be limited by the valve anatomy in patients with a large coaptation gap. Another mechanism to decrease TR in such patients involves the insertion of spacer devices (like the FORMA™ system) through the annulus over a railing device anchored to the right ventricle (Table 2). TriPair™ (Coramaze) is another spacer device that is being tested in the pre-clinical models; a retrievable atraumatic right atrium anchor and absence of a rail distinguish it from the FORMA™ system.

**Table 2:** Descriptive analysis of the different Transcatheter Tricuspid Valve Repair devices with human experience.

**Device**	**Mechanism**	**Specific characteristics**	**Level of evidence**
TriClip™ (Abbott)	Edge-edge repair	Based on Mitraclip™ technology. Most common repair device used to date. Trans-femoral access.	TRILUMINATE trial- feasibility study of 85 patients in patients with moderate or greater symptomatic TR with poor surgical candidacy– durable reduction in TR (71%) and reverse RV remodeling noted at 1-year (54). A randomized trial comparing TriClip with medical therapy in patients with severe TR at high surgical risk is currently ongoing (TRILUMINATE Pivotal) (55).
PASCAL™ (Edwards Lifesciences)	Edge-edge repair	A similar mechanism to the Triclip™. Trans-femoral access. A unique spacer helps bridge large coaptation gaps and reduces mechanical stress on the leaflets.	CLASP-TR: Feasibility study included 34 patients with severe or greater symptomatic TR at high surgical risk. The device was implanted successfully in 85% of the patients with durable reduction in TR at 30-days in 85% of those patients (56). CLASP-II TR: Ongoing randomized trial comparing tricuspid valve repair (PASCAL) with medical therapy (57).
Cardioband™ (Edwards Lifesciences)	Direct Annuloplasty	Cardioband™ is delivered through transfemoral access into the annulus and is positioned using up to 17 anchors. Once optimally positioned, it is contracted to decrease the tricuspid annulus. The right coronary artery can be affected by device contraction.	Initial European feasibility study (TRI-REPAIR) showed good technical success (100%) in 30 patients with moderate or higher symptomatic TR with favorable results at 2-year follow-up (58). An early feasibility study in the US enrolled 30 patients with severe or greater symptomatic functional TR with technical success in 93% of the patients and promising 30-day outcomes (59).
Tricinch™ (4Tech Cardio Ltd.)	Direct annuloplasty	A two-component device using trans-femoral access– a nitinol corkscrew implant is anchored on the AP tricuspid annulus which is coupled using a Dacron band with a self-expanding nitinol stent that is deployed in the IVC to maintain tension on the system and reduce annular dimensions. Given the valve and sub-valvular apparatus are intact, other therapies can be combined with Tricinch™.	An early feasibility study (PREVENT) in 15 symptomatic patients with moderate-severe TR with annular dilatation was terminated per the sponsor. In the TriValve Registry, 14 patients (4% of the patients) underwent tricuspid valve repair with Tricinch? with procedural success in 62.5% of the patients and no 30-day mortality. Patients with higher regurgitant volume in the registry underwent TTVr with Tricinch™ (60).
Trialign™ (Mitralign Inc.)	Direct annuloplasty	Trans jugular-based suture-based device that reduces tricuspid annular diameter by plication obliterating the posterior leaflet– replicates the surgical “Kay” procedure. Based on the Mitralign? device designed for MR. A guide catheter is used to engage the right coronary artery given its proximity to the annulus.	Early feasibility study (SCOUT) in 15 patients with moderate or greater functional TR showed good technical success (100% at the time of procedure, 80% at 30-days due to single-pledget annular detachments in 3 patients) with safety (61). A larger study to assess the safety and performance of Trialign™ in 60 patients with at least moderate functional TR across the United States and Europe is currently enrolling (62).
Mistral™ (Mitralix Ltd.)	Grasping of the chordae tendineae	Spiral-shaped, nitinol-device delivered transfemorally to grasp the chordae tendineae like a bouquet–this improves leaflet coaptation and improves RV geometry as well– dual mechanisms to decrease functional TR. This device further spares the valve leaflets; hence, other repair devices can still be used in cases of persistent TR.	First-in-human study in 7 patients with severe or greater TR at high surgical risk underwent successful tricuspid repair with the Mistral™ device with good efficacy results and improved RV function at 30-day follow-up (63).
FORMA™ (Edwards Lifesciences)	Spacer device	A foam-filled balloon (spacer- 12/15/18 mm) is positioned across the tricuspid valve over a rail extending from the subclavian vein to the RV apex. The device is anchored to the RV myocardium using a nitinol anchor with six prongs. There is a risk of endocarditis with the implanted device and the subcutaneous pocket. With anchoring of the device in the RV, the risk for perforation exists as well.	First-in-human experience in 19 patients with severe functional TR in Europe and Canada, showed feasibility with sustained TR reduction and functional improvement at 3-year follow-up. Device thrombosis and pulmonary embolism were notable adverse events with sub-therapeutic anticoagulation (64). Similar results were noted with 30-day follow-up in the US with 18 compassionate cases and 29 patients included in the early feasibility study. Two procedural failures in both groups related to perforation and device dislocation (65).
Caval Implantation (CAVI)	Heterotopic valve implantation	Bio-prosthetic valves implanted in the IVC and SVC to allow forward flow into the right atrium but no backflow during TR. The initial experience involved non-dedicated valves (Edwards Sapien™) while novel self-expandable valves dedicated for the bi-caval anatomy include the TricValve™ (P&F Products; CE approval), the Tricento™ (NewValve Technology) and the Trillium™ systems (66). The procedure can be performed without general anesthesia. Device use is limited in patients with severe RV failure, IVC diameter >45 mm, severe pulmonary hypertension, or contraindication to anticoagulation.	TRICAVAL compared medical therapy with CAVI in 28 patients with severe, symptomatic TR at high-surgical risk with Edwards SAPIEN XT balloon-expandable valve (23/26/29 mm). Patient recruitment was stopped early due to four complications within 48 h of the implant– two patients with tamponade due to stent migration and two valve dislocations. Further, no significant difference was noted in the maximal oxygen uptake or functional outcomes between the groups at 3-month follow-up (67). An early feasibility study for TricValve™ (TRICUS) and a CE mark trial (TRICUS-EURO) are currently ongoing (68).

*TR, Tricuspid Regurgitation; AP, Anteroposterior; IVC, Inferior Vena Cava; TTVr, Transcatheter Tricuspid Valve Repair; IVC, Inferior Vena Cava; SVC, Superior Vena Cava; CE, Conformité Européenne*.

In patients with large coaptation gaps (>6–8 mm), massive/torrential TR, device-related TR, or prior failed repair with single leaflet detachment, TTVr may not be feasible and this has created the niche for Caval Valve Implantation (CAVI) and Transcatheter Tricuspid Valve Replacement (TTVR). CAVI involves placement of a heterotopic valve in the Inferior Vena Cava and possibly the Superior Vena Cava (SVC) to protect the hepatic and renal circulation by redirecting the TR jet and this modality also improves the forward stroke volume ejected through the right ventricle. CAVI is technically easier than other transcatheter tricuspid therapeutic options and can be easily combined with other modalities as the valve or sub-valvular apparatus are intact. While the initial experience with non-dedicated valves showed a high rate of complications related to device thrombosis and dislocation, the novel dedicated bicaval valves are currently undergoing early feasibility studies (Table 2) (66). For TTVR, CardioValve™ (CardioValve Ltd.), EVOQUE™ (Edwards Lifesciences), Lux-Valve™ (Jenscare Biotechnology), NaviGate™ (NaviGate Inc.), Trisol™ (Trisol Medical), Intrepid™ (Medtronic), TRiCares™ (TRiCares SAS) are currently being tested in early feasibility trials (69). Most of these devices are deployed through transfemoral access except for Trisol™ needing transjugular access and minimally invasive right thoracotomy is needed for Lux-valve™ and Navigate™. Increased risk for prosthetic valve thrombosis due to a low-pressure system within the right heart needing anticoagulation, right-sided heart failure due to near-complete elimination of TR resulting in afterload mismatch, endocarditis, and conduction disturbances are some of the factors to be considered when deciding between transcatheter tricuspid valvular replacement vs. repair in a patient with difficult anatomy and future trials would further help develop an individualized approach for management of functional TR.

### Aortic Stenosis

In patients with severe Aortic Stenosis (AS) and left ventricular dysfunction or in patients with Continuous-Flow Left Ventricular-Assist Devices (CF-LVAD) who develop moderate or higher degrees of Aortic Insufficiency (AI), the role of transcatheter aortic valve interventions has been growing. In the TOPAS-TAVI registry, 293 patients with low-flow, low-gradient aortic stenosis and depressed ejection fraction were included and patients with severely depressed EF (<30%) had greater improvements in LVEF at 1-year follow-up after Transcatheter Aortic Valve Replacement (TAVR) compared to patients with moderately reduced EF (<40%), irrespective of the presence of contractile reserve with dobutamine stress (70). Aortic stenosis and left ventricular dysfunction commonly co-exist and it is not surprising to see improvement in cardiomyopathy once the afterload imposed by aortic stenosis is improved with TAVR. TAVR-UNLOAD is an ongoing, randomized controlled trial that would help assess if early TAVR with medical therapy improves outcomes compared to medical therapy alone outcomes in patients with moderate AS and HFrEF (71).

LVAD is increasingly being used as destination therapy in patients with advanced heart failure who are ineligible for heart transplantation. At the time of LVAD implantation, a moderate or higher degree of aortic regurgitation is commonly managed with complete patch closure, central oversewing of the aortic leaflets (Park's stitch), or replacement with a bio-prosthetic valve. Complete valve closure can be fatal in cases of device malfunction and the potential for myocardial recovery should also be weighed in. Surgical Aortic Valve Replacement (SAVR) at the time of VAD surgery would increase the bypass and aortic cross-clamping times and is associated with worse clinical outcomes in patients with INTERMACS 1 and 2 level heart failure (72). There is a role for TAVR in such patients. Further, the altered non-physiological flow profile with LVAD (particularly CF-LVAD) promotes aortic valve closure and commissural fusion leading to the development of *de-novo* AI following LVAD implantation– noted in ~15–52% of patients at 1-year post-implant (72). Moderate or higher degrees of AI leads to decreased cardiac output due to redundant backflow to the left ventricle and causes persistent heart failure in these patients. Given higher mortality rates from redo surgical repair or replacement due to severe co-morbidities, transcatheter treatment options are being used increasingly in these patients to treat significant post-LVAD AI. Occluder devices (Amplatzer™) have been used successfully in these patients, however, similar to complete surgical closure, these devices make the patient completely dependent on the LVAD (73). In a case series of 9 such patients, TAVR was used successfully with a self-expanding prosthesis resulting in a durable reduction of AI at 6-months. Owing to lack of calcium in pure AI lesions, the prosthesis is prone to migration– 2/9 patients needed implantation of a second valve due to device migration. Dedicated transcatheter valvular designs for AI with a “clasping” mechanism to facilitate anchoring (JenaValve™ and J-valve™) may help bridge this gap (74).

## Structural Interventions That Assist in Lowering Left-Atrial Pressure

Compared to the multitude of medical and device therapy options with a mortality benefit for HFrEF, limited options prevail for the management of patients with HFpEF. Patients with Lutembacher syndrome– congenital or acquired mitral stenosis and an atrial septal defect do not suffer from congestive symptoms. This inspired the development of Transcatheter Interatrial Shunt Devices (IASD)– these devices are implanted through the femoral veins and trans-septal access and aim to lower the left-atrial pressure with activity thereby improving symptoms and outcomes in patients with HFpEF and HFmEF. REDUCE-LAP HF I was a phase-II randomized trial in patients with symptomatic refractory heart failure (wedge pressure ≥25 mm Hg during exercise) with LVEF>40% and a gradient of ≥5 mm Hg between the left and right atria– patients were randomized to treatment with IASD (Corvia^®^ Atrial Shunt) vs. a sham control and significant reduction in pulmonary wedge pressure with exercise was noted in patients treated with device therapy at 1-month follow-up (75). Further, longer follow-up at 1 year showed patency of the shunts with no significant adverse outcomes (76). However, REDUCE-LAP HF II– a randomized controlled trial comparing treatment with IASD (Corvia^®^ Atrial Shunt) vs. a sham control in a similar heart failure population failed to show any improvement in heart failure events or heart failure symptoms at 12–24 months follow-up (77). Patients with right-sided dysfunction, pulmonary hypertension, valvular heart disease, recent deep vein thrombosis or pulmonary embolism, or stroke are not candidates for this device therapy. Interestingly, in REDUCE-LAP HF II trial, the only sub-group that benefited from IASD included patients without evidence of latent pulmonary vascular disease suggesting a possible role of invasive exercise hemodynamics for optimal patient selection (77). While Corvia^®^ Atrial Shunt is a valveless self-expandable device with a double-disc design with an 8-mm opening in the center for an optimal inter-atrial shunt, the V-wave Ventura^®^ IASD is an hourglass-shaped device that included a one-way porcine tissue valve in the initial versions– in the first-in-human study of 38 patients with HFrEF and HFpEF, it was feasible and safe but was associated with poor long-term shunt patency rates (36% at 12-months) (78). A modified valveless version of the V-wave Ventura^®^ IASD with an internal diameter of 5 mm is currently being investigated in a randomized controlled trial including patients with symptomatic heart failure, irrespective of the LVEF- RELIEVE-HF trial (79). Another device- the Atrial Flow Regulator (AFR, Occlutech)– is a self-expandable nitinol device that needs balloon septal dilation before device deployment and has two different shunt sizes (8 and 10 mm) based on the left-sided filling pressures– the pilot study (AFR-PRELIEVE) showed safety, feasibility and good patency rates at 3-months follow-up (80).

Recently, there has been active research in the development of implant-free Inter-Atrial Shunts– the Alleviant System is one such strategy that creates a shunt using radiofrequency energy-based septectomy that has shown safety and clinical efficacy with patency through 12-months follow-up in the first-in-human clinical study (ALLEVIATE HF-1) (81). The implant-free approach has a unique advantage over implantable IASDs–it does not preclude the use of the inter-atrial septum for any future structural or electrophysiological interventions.

## Structural Interventions for Hemodynamic Monitoring

Implantable micro-electromechanical-based sensors in the Pulmonary Artery (PA) have been developed to assist in real-time monitoring of cardiac filling pressures to tailor medical therapy and pre-empt a heart failure exacerbation. This is particularly important for patients with HFpEF, where patients are threading a narrow line between hypervolemia and heart failure and hypovolemia and underfilling of the ventricle resulting in hypotension. CHAMPION was a single-blinded randomized controlled trial that showed a clinically meaningful and significant reduction in HF admissions in patients with moderately symptomatic heart failure (New York Heart Association (NYHA) class III HF with a recent admission in the past 12 months) with the use of the wireless PA pressure monitoring using the CardioMEMS™ HF system (Abbott) compared to medical therapy alone. HF admissions were consistently reduced in patients with HFpEF and HFrEF with a significant change in diuretic dosing across the two arms; this effect was apparent after about 3 months of diuretic titration in the treatment arm and persisted up to 17 months of follow-up (50% relative reduction for HFpEF and 26% for HFrEF) (82). CardioMEMS™ HF system was approved by the Food and Drug Administration for the indications studied in the CHAMPION trial in 2014. Recently, the GUIDE-HF trial studied hemodynamic monitoring-guided management of HF with the CardioMEMS™ HF system compared to medical therapy alone in patients with mild-severe chronic HF (NYHA class II-IV) and patients were not required to have a recent HF admission if they had elevated natriuretic peptides– no significant difference was noted in the primary composite end-point of all-cause mortality and HF events in either HFrEF or HFpEF at 12 months follow-up in this trial (83). It is important to note that the disruptions caused by the Coronavirus Disease 2019 (COVID-19) pandemic may have had a significant impact on this trial results– analyzing the pre-specified pre-COVID-19 sub-group, a benefit was noted with reduced HF admissions in the hemodynamic monitoring-guided management arm, however, it is hypothesis-generating as this analysis lacks adequate power. Another similar micro-electromechanical-based sensor is the Cordella™ (Endotronix Inc.) device that showed promising safety and accuracy data (SIRONA first-in-human study) and is currently being studied in a randomized controlled trial in patients with NYHA class III HF (PROACTIVE-HF) (84, 85). Compared to the CardioMEMS™ HF system that is implanted in the left pulmonary artery, the Cordella™ HF system is implanted in the right PA and the integrated system includes the incorporation of clinical variables in the form of symptoms heart rate, blood pressure, oxygen saturation and weight in addition to the invasive hemodynamic data for HF management. While helpful in monitoring left-sided filling pressures, these devices offer little in terms of right-sided pressure monitoring and use can be limited in patients with recent pulmonary embolism or a predisposition to recurrent pulmonary emboli.

## Ventricular Restorative Devices

In HFrEF patients, with disease progression, the left ventricle remodels into a dilated, spherical cavity to compensate and maintain cardiac output based on the Frank-Starling relationship. However, this adverse remodeling results in increased end-diastolic volumes with increased wall stress, more FMR, and drives refractory heart failure symptoms. Ventricular restorative devices are being actively researched to restore the altered LV geometry in patients with refractory heart failure. Parachute^®^ (Cardiokinetix) is a catheter-based, self-expanding, umbrella-shaped, partitioning device, that was intended to separate the aneurysmal portion of the LV and create a new apex in patients with ischemic cardiomyopathy. While the first-in-human study (PARACHUTE) in patients with HFrEF (EF 15–40% and an akinetic of dyskinetic apex) showed safety and feasibility, at 3-year follow-up, a reduction in stroke volume and LVEF were noted. This device was subsequently tested in a randomized, controlled trial (PARACHUTE IV) that enrolled 331 subjects with NYHA class III-IV ischemic cardiomyopathy and wall motion abnormalities (EF 15–35%) with suitable anatomy for the device, however, the trial was terminated prematurely in 2017 and this device is not in use currently (86). Another device designed to help restore the dysfunctional geometry in a failing ventricle is the AccuCinch^®^ (Ancora Heart Inc.) Ventricular Restoration System– a nitinol anchor-based cinching cable positioned in the LV cavity below the mitral valve through a retrograde transventricular approach. This is still being tested in early feasibility trials in patients with HFrEF or moderate or higher degree of FMR with NYHA class III-IV symptoms despite optimal medical therapy (CorCinch) (87). BioVentrix Revivent TC™ is another alternative to surgical left ventricular reconstruction. This device is implanted through a hybrid mini-thoracotomy (left ventricle-septum-right ventricle) and transcatheter approach (neck vein-right ventricle) with its internal anchor implanted into the right ventricle across the anterior septum and the external anchor latched onto the epicardial surface– it restores left ventricular geometry in patients with ischemic cardiomyopathy through the exclusion of scarred/dyskinetic/aneurysmal myocardium. A single-arm multi-center study of 86 patients with symptomatic ischemic cardiomyopathy (LVEF 15–45%) showed sustained improvement in heart failure symptoms, LVEF and LV volumes at 12 months with this device with >90% survival (4 in-hospital deaths were noted) (88). Based on these promising results, this device has been granted CE approval in Europe and is currently being evaluated against medical therapy in the ongoing pivotal ALIVE trial (89).

## Cardiac Soft-Robotic Sleeves

Cardiac sleeves envisage the use of biomimetic implantable robotic devices for ventricular support in heart failure patients. The sleeves have certain benefits compared to current VAD in clinical practice– they can provide bi-ventricular support, no contact with blood reduces the risk for thrombosis, they can be activated to provide support to the heart during systole as well as diastole, and finally, level of support can be weaned as the native function recovers (90). These robotic sleeves are still being tested in pre-clinical animal models. CorInnova Inc has developed such a pneumatically actuated robotic device for biventricular support with a polyurethane membrane on a self-expanding nitinol frame that can be deployed through a mini-thoracotomy with a sutureless pneumatic attachment to the heart– it has shown promising results in pre-clinical ovine models with the first-in-human studies are being planned (91). This device is intended as a short-term cardiac assist device as bridge-to-decision or bridge-to-transplant and for long-term use in patients with advanced heart failure who are ineligible for VADs.

## Conclusion

In summary, in the last decade, there has been tremendous growth in the development of transcatheter interventions to improve heart failure outcomes. Given the multitude of structural therapies available and the clinical complexity involved in decision-making, the role of team-approach with involvement of the advanced heart failure team, multi-modality cardiac imagers, different medical sub-specialties (intensive care, nephrology and hepatology) and the structural heart team cannot be overstated. Finally, early consideration of structural therapies is paramount to ensure good outcomes before end-organ damage has ensued. The ongoing trials for cardiac robotic sleeves, ventricular restorative devices, implant-free inter-atrial shunts, novel transcatheter mitral and tricuspid valve devices would help shape the future of heart failure management.

## Author Contributions

DK and MM were involved in the planning, execution, writing, and critical appraisal of this manuscript. All authors contributed to the article and approved the submitted version.

## Conflict of Interest

MM: primary investigatory at University of Miami for Clinical Evaluation of the AccuCinch ventricular restoration system in patients who present with symptomatic heart failure with reduced ejection fraction (HFrEF): the CORCINCH-HF study. The remaining author declares that the research was conducted in the absence of any commercial or financial relationships that could be construed as a potential conflict of interest.

## Publisher's Note

All claims expressed in this article are solely those of the authors and do not necessarily represent those of their affiliated organizations, or those of the publisher, the editors and the reviewers. Any product that may be evaluated in this article, or claim that may be made by its manufacturer, is not guaranteed or endorsed by the publisher.
